# Plasmid Dynamics of *mcr-1*-Positive *Salmonella* spp. in a General Hospital in China

**DOI:** 10.3389/fmicb.2020.604710

**Published:** 2020-12-22

**Authors:** Jianzhong Fan, Linghong Zhang, Jintao He, Maoying Zhao, Belinda Loh, Sebastian Leptihn, Yunsong Yu, Xiaoting Hua

**Affiliations:** ^1^Department of Laboratory Medicine, Affiliated Hangzhou First People’s Hospital, Zhejiang University School of Medicine, Hangzhou, China; ^2^Department of Infectious Diseases, Sir Run Run Shaw Hospital, Zhejiang University School of Medicine, Hangzhou, China; ^3^Key Laboratory of Microbial Technology and Bioinformatics of Zhejiang Province, Hangzhou, China; ^4^Regional Medical Center for National Institute of Respiratory Diseases, Sir Run Run Shaw Hospital, School of Medicine, Zhejiang University, Hangzhou, China; ^5^Department of Laboratory Medicine, Hangzhou Hospital of Traditional Chinese Medicine, Hangzhou, China; ^6^Zhejiang University-University of Edinburgh (ZJU-UoE) Institute, Zhejiang University, Haining, China

**Keywords:** *Salmonella*, *mcr-1*, IS, inactivation, IS*Apl1*, IS*Vsa5*

## Abstract

*Salmonella* is an important food pathogen that can cause severe gastroenteritis with more than 600,000 deaths globally every year. Colistin (COL), a last-resort antibiotic, is ineffective in bacteria that carry a functional *mcr-1* gene, which is often spread by conjugative plasmids. Our work aimed to understand the prevalence of the *mcr-1* gene in clinical isolates of *Salmonella*, as the frequency of occurrence of the *mcr-1* gene is increasing globally. Therefore, we analyzed 689 clinical strains, that were isolated between 2009 and late 2018. The *mcr-1* gene was found in six strains, which we analyzed in detail by whole genome sequencing and antibiotic susceptibility testing, while we also provide the clinical information on the patients suffering from an infection. The genomic analysis revealed that five strains had plasmid-encoded *mcr-1* gene located in four IncHI2 plasmids and one IncI2 plasmid, while one strain had the chromosomal *mcr-1* gene originated from plasmid. Surprisingly, in two strains the *mcr-1* genes were inactive due to disruption by insertion sequences (ISs): IS*Apl1* and IS*Vsa5*. A detailed analysis of the plasmids revealed a multitude of ISs, most commonly IS*26*. The IS contained genes that meditate broad resistance toward most antibiotics underlining their importance of the mobile elements, also with respect to the spread of the *mcr-1* gene. Our study revealed potential reservoirs for the transmission of COL resistance and offers insights into the evolution of the *mcr-1* gene in *Salmonella*.

## Introduction

Colistin (COL) is a polypeptide antibiotic that was first isolated from the supernatant of a *Bacillus polymyxa var. colistinus* culture (Tambadou et al., 2015). It has been used in both human and veterinary medicine for more than 50 years, although the parenteral use in humans is limited due to issues with nephrotoxicity and neurotoxicity (Landman et al., 2008; Biswas et al., 2012). Due to the increase of antimicrobial resistance and the lack of antibiotic compounds that are effective, COL can be deployed in the clinic in combination with other drugs that protect renal function. At present, COL is considered as the last resort option for the treatment of infections caused by Gram-negative bacteria that are multidrug-resistant (MDR), extensively drug-resistant (XDR), or pandrug-resistant (PDR) (Nation and Li, 2009).

In 2015, Liu et al. first discovered plasmid-encoded COL resistance, mediated by the gene *mcr-1*, in *Escherichia coli* isolated from animals, and demonstrated that the gene, encoded on conjugative plasmids, can be used by different strains to mediate low levels of COL resistance (Liu et al., 2016). While the acquisition of the *mcr-1* gene does not result in a new bacterial strain, the recipient strain develops resistance to COL (Schwarz and Johnson, 2016). As the *mcr-1* gene is highly transmissible, it has been observed in more than 30 countries on six continents (Wang et al., 2019; Shen et al., 2020), and can be found in different genera (such as *E. coli*, *Klebsiella pneumoniae*, *Shigella sonnei*, and *Salmonella*) isolated from animals, food, or humans worldwide (Hu et al., 2016; Liu et al., 2016; Pham Thanh et al., 2016; Schwarz and Johnson, 2016; Al-Tawfiq et al., 2017; Zhang et al., 2019). Strains of *Salmonella* are important pathogens of concern in food safety, as they are frequently transmitted between agricultural animals, food, and humans (Foley and Lynne, 2008); the pathogens often cause gastroenteritis, in some cases severe, and are responsible for >600,000 deaths annually (Lokken et al., 2016). Increasing antimicrobial resistance in *Salmonella* species is considered an important public health concern of the 21st century (Lozano-Leon et al., 2019). *Salmonella* strains that acquire multidrug resistance genes would be difficult to treat and would result in an even higher number of cases with severe morbidity and high frequency of mortality. After *Salmonella* species carrying *mcr-1* were detected in the United Kingdom in 2016, the level of occurrence of *mcr-1* in *Salmonella* among livestock and humans, but also the environment, is increasing worldwide (Cui et al., 2017; Yi et al., 2017b). In addition, although another five *mcr* variants (*mcr-2*, *mcr-3*, *mcr-4*, *mcr-5*, and *mcr-9*) have been identified in *Salmonella* species (Carattoli et al., 2018; Garcia-Graells et al., 2018; Borowiak et al., 2019; Carroll et al., 2019; Sun et al., 2020), *mcr-1* is still the most common *mcr* gene found in COL-resistant *Salmonella* spp. (Borowiak et al., 2020).

Many studies have focused on the *mcr-1* gene-carrying *E. coli* and *K. pneumonia* strains, while the prevalence and molecular characteristics of the *mcr-1* gene in *Salmonella* spp. have been investigated in much less detail. The goal of our study is to understand the prevalence of the human-derived *mcr-1* gene in community-acquired *Salmonella* infections in clinical isolates. We conducted a retrospective study to determine the prevalence of *mcr-1* positive *Salmonella* in 689 clinical strains that were isolated between 2009 and 2018. To analyze the level of drug-resistance, we also tested the sensitivity of the isolates to different antibiotic classes, other than COL, and performed a detailed genomic analysis of all *mcr-1* positive strains.

## Materials and Methods

### Strains Source

*Salmonella* clinical strains were collected from patients in the First People’s Hospital of Hangzhou, Zhejiang Province, China between 2009 and 2018. Most specimens were collected from the departments of Pediatrics, Internal Medicine, Gastroenterology Department and Infectious Medicine while other departments were only a secondary source for the isolates. The specimen types of clinical isolates collected included blood, feces, and pus. The cultured bacteria were stored in glycerol broth at −80°C. The DNA from isolates were extracted and screened for the *mcr-1* gene by PCR. The *mcr-1*-positive isolates were verified by Sanger sequencing. This study was approved by the Ethics Committee of Hangzhou First People’s Hospital (2020103-1) with a waiver of informed consent because of the retrospective nature of the study. All strains were identified using the automated Vitek 2 system (BioMérieux, Marcy-l’Étoile, France) and MALDI-TOF MS (Bruker, Bremen, Germany). *Salmonella* serotyping was conducted by slide agglutination with specific antisera (Tianrun Bio-Pharmaceutical Co., Ltd., Ningbo, China) according to the White-Kauffmann-Le Minor scheme (9th edition).

### PCR Amplifications and Sequencing

All collected isolates were screened for the presence of *mcr-1* positive strains using PCR with the primers *mcr-1*-forward (5′-GCTCGGTCAGTCCGTTTG-3′) and *mcr-1*-reverse (5′-GAA TGCGGTGCGGTCTTT-3′). The amplicons were subsequently sequenced by Sanger sequencing (Quan et al., 2017).

### Antimicrobial Susceptibility Test

Broth micro-dilution and *E*-test method were used to determine the minimal inhibitory concentrations (MICs) of COL and 13 other antibiotics: ampicillin (AMP), ceftriaxone (CRO), cefepime (FEP), tetracycline (TET), amoxicillin/clavulanic acid (AMC), cefotaxime (CTX), chloramphenicol (C), meropenem (MEM), imipenem (IPM), azithromycin (AZM), cefoxitin (FOX), amikacin (AK) and ciprofloxacin (CIP), according to the Clinical and Laboratory Standards Institute (CLSI) M100-S29 (CLSI, 2019). Most results were interpreted in accordance with CLSI, except COL and tigecycline, for which we used the European Committee on Antimicrobial Susceptibility Testing (EUCAST) breakpoints v8.1. The quality control strain used in the antimicrobial sensitivity test was *E. coli* ATCC 25922.

### Genomic DNA Sequencing and Bioinformatics Analysis

Genomic DNA was extracted from six *mcr-1*-positive strains and sequenced using the Illumina HiSeq and Nanopore MinION platforms. Long-read library preparation for Nanopore sequencing was performed with a 1D sequencing kit (SQK-LSK109; Nanopore) without fragmentation. The libraries were then sequenced on a MinION device with a 1D flow cell (FlO-MIN106; Nanopore) and base called with Guppy v2.3.5 (Nanopore). The long read and short read sequence data were used in a hybrid *de novo* assembly using Unicycler v0.4.8 (Wick et al., 2017), then polished with Pilon v1.23 (Walker et al., 2014). Antibiotic resistance genes were identified using the ResFinder database (Zankari et al., 2012) with Abricate 0.8^1^ or BacAnt (Hua et al., 2020). Multi-locus sequence typing (MLST) was performed using mlst^2^. Sequence comparisons were performed using BLAST and visualized with Easyfig 2.2.2 (Sullivan et al., 2011).

### Plasmid Conjugation Experiments

Conjugation experiments were performed by broth and filter mating using the sodium azide-resistant *E. coli* J53 as the recipient strains. The mixture (ratio of 1:1) of donors and the recipient strain J53 were subjected to incubation on MH agar plates for 18 h at 37°C. The successful transconjugants were selected on MH agar plates supplemented with 250 μg/mL sodium azide and 2 μg/mL CTX (or 1 μg/mL COL). The carriage of such a plasmid in the parental strain and the corresponding transconjugants were confirmed by PCR and MALDI-TOF MS. The MIC profiles of the transconjugants were also determined with antibiotics (AK, CTX, COL, TET, and AMP) by the broth microdilution method (CLSI, 2019).

## Results

### Screening for *mcr-1* Positive Strains

A total of 689 clinical *Salmonella* isolates were screened in this study, all of which were derived from patient specimen isolates from May 2009 to December 2018 in Hangzhou First People’s Hospital, Zhejiang Province, China. The isolates analyzed included more than ten species of *Salmonella*, that cause a wide range of pathologies including typhoid fever, swine cholera, and enteritis and belong to various *Salmonella* spp. such as *S.* Typhimurium, *S.* Enteritidis, *S.* Choleraesuis, *S.* Thompson, *S.* Manhattan, *S.* Derby, *S.* London, *S.* Senftenberg, and *S.* Aberdeen (Table 1). When screening for the *mcr-1* gene by PCR, we detected six isolates (0.87%) of the 689 *Salmonella* strains, that were *mcr-1* positive. Interestingly, the isolate S520 showed a longer *mcr-1* PCR product than other strains (Supplementary Figure 1), indicating an insertion in the *mcr-1* gene, or possibly a duplication. To confirm that these six strains harbored the *mcr-1* gene, we performed whole genome sequencing. While five strains had a plasmid-encoded *mcr-1* gene, one strain contained the gene as part of the bacterial chromosome. The results are described below in more detail.

**TABLE 1:** Serotype distribution of 679 *Salmonella* isolates.

Serogroup	Serotype	O antigens	H antigens	No. of isolates (%)
A group (2)	*S.* Paratyphi A	1, 2, 12	a: [1, 5]	2 (0.3)
B group (289)	*S.* Typhimurium	1, 4, [5], 12	i: 1, 2	227 (33.0)
	*S.* Derby	1, 4, [5], 12	f, g: [1, 2]	20 (2.9)
	*S.* Saintpaul	1, 4, [5], 12	e, h: 1, 2	9 (1.3)
	*S.* Agona	1, 4, [5], 12	f, g, s: [1, 2]	12 (1.7)
	*S.* Stanleyville	1, 4, [5], 12, [27]	d: 1, 2	7 (1.0)
	*S.* Indiana	1, 4, 12	z: 1, 7	4 (0.6)
	others	1, 4, 12	–	10 (1.5)
C1 group (118)	*S.* Choleraesuis	6, 7	c: 1, 5	12 (1.7)
	*S.* Infantis	6, 7, 14	r: 1, 5	18 (2.6)
	*S.* Irumu	6, 7	l, v: 1, 5	5 (0.7)
	*S.* Virchow	6, 7, 14	r: 1, 2	5 (0.7)
	*S.* Thompson	6, 7, 14	k: 1, 5	27 (3.9)
	*S.* Potsdam	6, 7, 14	l, v: e, n, z15	10 (1.5)
	*S.* Braenderup	6, 7, 14	e, h: e, n, z15	7 (1.0)
	*S.* Mbandaka	6, 7, 14	z10: e, n, z15	4 (0.6)
	*S.* Rissen	6, 7, 14	f, g: –	5 (0.7)
	*S.* Montevideo	6, 7, 14	g, m, [p], s: [1, 2, 7]	2 (0.3)
	others	6, 7, 14	–	23 (3.3)
C2 group (35)	*S.* Manhattan	6, 8	d: 1, 5	3 (0.4)
	*S.* Newport	6, 8, 20	e, h: 1, 2	7 (1.0)
	*S.* Bovismorbificans	6, 8, 20	r, [i]: 1, 5	10 (1.5)
	*S.* Litchfield	6, 8	l, v: 1, 2	9 (1.3)
	others	6, 8	–	6 (0.9)
D group (157)	*S.* Enteritidis	1, 9, 12	g, m: [1,7]	154 (22.4)
	*S.* Gallinarum-pullorum	1, 9, 12	–	2 (0.3)
	others	1, 9, 12	:H5	1 (0.1)
E1 group (51)	*S.* London	3, {10} {15}	l, v: 1, 6	37 (5.4)
	*S.* Weltevreden	3, {10} {15}	r: z6	5 (0.7)
	*S.* Ruzizi	3, 10	l, v: e, n, z15	1 (0.1)
	*S.* Vejle	3, {10} {15}	e, h: 1, 2	1 (0.1)
	*S.* Anatum	3, {10} {15} {15,34}	e, h: 1, 6	1 (0.1)
	others	10	–	6 (0.9)
E4 group (25)	*S.* Senftenberg	1, 3, 19	g, [s], t: –	21 (3.0)
	others	19	–	4 (0.6)
F group (5)	*S.* Aberdeen	11	i: 1, 2	5 (0.7)
Other groups (5)	*S. enterica* subsp. *diarizonae*			2 (0.3)
	others			3 (0.4)

### Clinical Information on Patients Suffering From a *Salmonella mcr-1* Positive Infection

The detailed clinical information about *mcr-1* positive patients is shown in Table 2. The oldest *mcr-1* positive strain was isolated in May 2015, from a 1-year-old male infant. The second oldest strain was isolated in 2016. Three strains have been obtained in 2017, and one strain in 2018. Four patients suffering from infections with a *mcr-1* positive strain were young children under 3 years, while the remaining two were isolated from elderly people (65 and 84 years old). All patients had symptoms of gastroenteritis, gastrointestinal dysfunction, or diarrhea. Among the six strains, five isolates (S304, S438, S441, S520, S585) belonged to *S.* Typhimurium/ST34 (O4:Hi), the remaining one (S530) to *S.* Indiana/ST17 (O4:Hz:H7).

**TABLE 2:** Basic situation of *mcr-1* positive strains.

Strain	Inspection time	Specimen type	Patient age	Patient sex	Diagnosis of infection	Visiting department	Serovar	Serotype	AMR phenotype
S304	2015.5	Stool	1	Male	Acute enteritis	Pediatrics	*S.* Typhimurium	O4:Hi:	COL, AMP, CRO, FEP, TET, AMC, CTX, FOX, C,
S438	2016.12	Stool	3	Male	Enteritis	Pediatrics	*S.* Typhimurium	O4:Hi:	COL, AMP, CRO, FEP, TET, AMC, CTX, C
S441	2017.3	Stool	3	Female	Infectious diarrhea	Pediatrics	*S.* Typhimurium	O4:Hi:	COL, AMP, CRO, AZM, FEP, TET, AMC, CTX, FOX, C
S520	2017.9	Stool	1	Female	Diarrhea	Pediatrics	*S.* Typhimurium	O4:Hi:	AMP, CRO, FEP, TET, AMC, CTX, CIP, AK, C
S530	2017.1	Stool	84	Female	Fracture, diarrhea	Recovery unit	*S.* Indiana	O4:Hz:H7	AMP, CRO, AZM, FEP, TET, AMC, CTX, CIP, AK, C
S585	2018.12	Stool	65	Female	Gastrointestinal disorders	Recovery unit	*S.* Typhimurium	O4:Hi:	COL, AMP, CRO, FEP, TET, AMC, CTX, C

*AMR, antimicrobial resistance; COL, colistin; AMP, ampicillin; CRO, ceftriaxone; AZM, aztreonam; FEP, cefepime; TET, tetracycline; AMC, amoxicillin/clavulanate; CTX, cefotaxime; FOX, cefoxitin; CIP, ciprofloxacin; AK, amikacin; IPM, imipenem; C, chloramphenicol; MEM, meropenem.*

### Drug Susceptibility of the *mcr-1* Positive Strains

Clinical strains that show COL resistance are often resistant to several antibiotics. We therefore tested the susceptibility of the isolates that were *mcr-1* positive toward several antibiotics applying the MIC standard values of the 2019 CLSI standard. The drug susceptibility results are shown in Table 2 and Supplementary Data Sheet 1. Surprisingly, only four of the six *mcr-1* positive strains were resistant to COL in various degrees (three up to 32 μg/mL, one 4 μg/mL), while the two isolates S520 and S530 were not resistant to COL, indicating that their *mcr-1* genes (or their regulation) might not be functional. All six strains displayed resistance to AMP, CRO, FEP, TET, AMC, CTX, and chloramphenicol, but were not resistant to MEM nor IPM. With the exception of strains S441 and S530, four strains were sensitive to AZM. Two isolates, S304 and S441, were resistant to FOX, while the others were sensitive to the compound. Four strains were sensitive to AK, while two (S520 and S530) were resistant to the antibiotic. Strains S304 and S438 were sensitive, two intermediate (S441 and S585) and two (S520 and S530) were resistant to CIP. The results of our antibiotic susceptibility testing show that most strains that carry a COL resistant gene are also resistant to many other antibiotics, and can thus be considered multidrug resistant, making treatment challenging.

### Genomic Location of *mcr-1* in the Six Isolates

**FIGURE 1:**
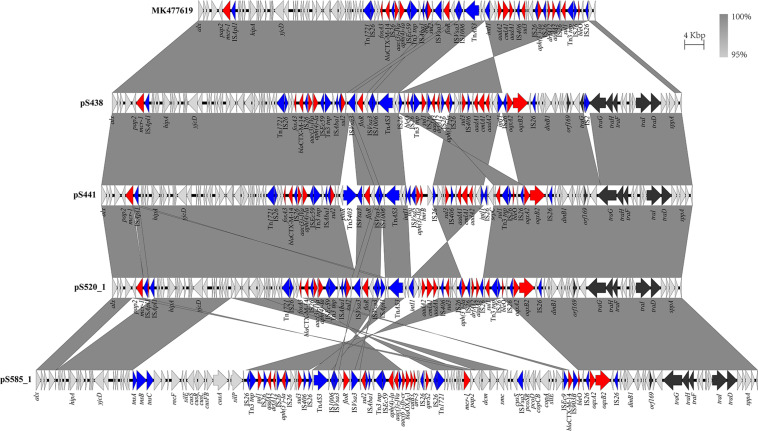
Scaled, linear diagrams comparing the sequences of plasmid pSH16G4918 (GenBank no. MK477619), pS438, pS441, pS520, and pS585_1. Antibiotic resistance genes are indicated in red arrows. The individual conjugation-related genes are indicated with black arrows. Blue arrows denote transposon- and integron-associated genes. Other genes are shown as gray arrows.

The genomic characteristics of the six isolates with regard to antimicrobial resistance are shown in Table 3. In four isolates (four *S.* Typhimurium), the *mcr-1* gene was located on derivatives of an IncHI2 plasmid (∼253–284 kb). All four IncHI2 plasmids belonged to sequence type 3 and were similar to a number of IncHI2 plasmids of *S.* Typhimurium from different origins including animals, food, and humans (Supplementary Figure 2). The genetic context of the *mcr-1* gene was relatively similar within these plasmids in three strains (S438, S441, S520), with the exception of strain S585. The *mcr-1* gene in S585 was located in variable region of IncHI2 plasmid without being embedded in an IS. In addition to the *mcr-1* gene, all four IncHI2 plasmids encoded other antibiotic resistance genes, including beta-lactam (*bla*_CTX–M–14_), aminoglycoside [*aac(3)-IVa*, *aadA2*, and *aph(4)-Ia*], sulphonamide (*sul1*, *sul2*, and *sul3*), phenicol (*floR*), quinolone (*oqxAB*), and fosfomycin (*fosA3*). A comparison of the plasmid sequences indicated many ISs and transposon insertion events, including IS*Apl1*, IS*186B*, IS*103*, IS*2*, Tn*5403*. Furthermore, our analysis revealed that IS*26* was responsible for a majority of inversion events (Figure 1). Plasmid pS438 contains 21 ISs, of which nine were IS*26* copies, two IS*Vsa3* copies, two IS*2* copies, and one copy each of IS*Kpn8*, IS*Ec59*, IS*Apl1*, IS*Aba1*, IS*Aba1*, IS*103*, IS*150*, and IS*1006*, respectively. An inversion of ca 23 kb fragment of nine antibiotic resistance genes including *aadA1*, *cmlA1*, *aadA1*, and *sul3* was observed in pS438 when compared to the pSH16G4918 (GenBank accession no. MK477619). In pS438, two copies of IS*26* adjacent to the 23 kb fragment are in opposite orientation and are flanked by an identical 8-bp repeat inverted relative to each other. This inversion was caused by the intramolecular transposition *in trans* of IS*26* into a target site.

**TABLE 3:** The summary of the features associated with the genome and plasmid identified in *Salmonella* genomes.

Sequence name	Size (bp)	ST type	Replicon Type (s)	Antibiotic resistance genes
S304	4,937,766	ST34		*bla*_TEM–1B_, *bla*_TEM–1B_, *aph(6)-Id*, *aph(3″)-Ib*, *sul2*, *aac(6*′*)-Iaa*, *mdf(A)*
pS304-1	254,873		IncHI2 IncHI2A	*aadA2*, *cmlA1*, *ant(3″)-Ia*, *sul3*, *aac(3)-IVa*, *aph(4)-Ia*, *aph(3*′*)-Ia*, *aph(6)-Id*, *aph(3″)-Ib*, *bla*_TEM–1B_, *tet(A)*, *qnrS1*, *ARR-3*, *cmlA1*, *bla*_OXA–10_, *ant(3″)-Ia*, *dfrA14*, *tet(A)*, *floR*, *bla*_CTX–M–65_
pS304_2	60.870		IncI2	*mcr-1.1*
pS304_3	7395		ColRNAI	
S441	4,944,769	ST34		*tet(B)*, *sul2*, *aph(3″)-Ib*, *aph(6)-Id*, *bla*_TEM–1B_, *qnrS1*, *bla*_CTX–M–55_, *aac(6*′*)-Iaa*, *mdf(A)*
pS441	260,707		IncHI2 IncHI2A	*mcr-1.1*, *fosA3*, *bla*_CTX–M–14_, *aac(3)-IVa*, *aph(4)-Ia*, *sul2*, *floR*, *aac(3)-IId*, *sul3*, *ant(3″)-Ia*, *cmlA1*, *aadA2*, *sul1*, *oqxA*, *oqxB*
S438	4,971,941	ST34		*bla*_TEM–1B_, *aph(6)-Id*, *aph(3″)-Ib*, *sul2*, *tet(B)*, *aac(6*′*)-Iaa*, *floR*, *sul2*, *aph(4)-Ia*, *aac(3)-IVa*, b*la*_CTX–M–14_, *fosA3*, *mdf(A)*
pS438	253,817		IncHI2 IncHI2A	*mcr-1.1*, *fosA3*, *bla*_CTX–M–14_, *aac(3)-IVa*, *aph(4)-Ia*, *sul2*, *floR*, *sul1*, *aadA2*, *dfrA12*, *aph(3*′*)-Ia*, *sul3*, *ant(3″)-Ia*, *cmlA1*, *aadA2*, *oqxA*, *oqxB*
S520	5,038,289	ST34		*aac(6*′*)-Iaa*, *mdf(A)*, *tet(B)*, *sul2*, *aph(3″)-Ib*, *aph(6)-Id*, *bla*_TEM–1B_
pS520	253,797		IncHI2 IncHI2A	*mcr-1.1*, *fosA3*, *bla*_CTX–M–14_, *aac(3)-IVa*, *aph(4)-Ia*, *sul2*, *floR*, *aadA2*, *cmlA1*, *ant(3″)-Ia*, *sul3*, *aph(3*′*)-Ia*, *dfrA12*, *aadA2*, *sul1*, *oqxA*, *oqxB*
S530	5,059,260	ST17	IncN IncQ1	*dfrA17*, *aadA5*, *aac(6*′*)-Ib-cr*, *bla*_OXA–1_, *catB3*, *ARR-3*, *sul1*, *fosA3*, *bla*_CTX–M–14_, *aac(3)-IVa*, *aph(4)-Ia*, *sul2*, *floR*, *mcr-1.1*, *mph(A)*, *sul3*, *tet(A)*, *bla*_TEM–1B_, *sul2*, *aph(3″)-Ib*, *aph(6)-Id*, *oqxA*, *oqxB*, *mph(E)*, *msr(E)*, *armA*, *sul1*, *ARR-3*, *catB3*, *bla*_OXA–1_, *aac(6*′*)-Ib-cr*, *aac(6*′*)-Iaa*, *mdf(A)*
S585	5,024,322	ST34		*mdf(A)*, *aac(6*′*)-Iaa*, *tet(B)*, *aph(6)-Id*, *aph(3″)-Ib*, *sul2*
pS585_1	284,303		IncHI2 IncHI2A	*sul1*, *aadA2*, *dfrA12*, *aph(3*′*)-Ia*, *sul3*, *floR*, *sul2*, *aph(4)-Ia*, *aac(3)-IVa*, *aac(6*′*)-Ib-cr*, *bla*_OXA–1_, *catB3*, *ARR-3*, *qnrS2*, *mcr-1.1*, *bla*_CTX–M–14_, *oqxA*, *oqxB*
pS585_2	6,079		ColRNAI	

**FIGURE 2:**
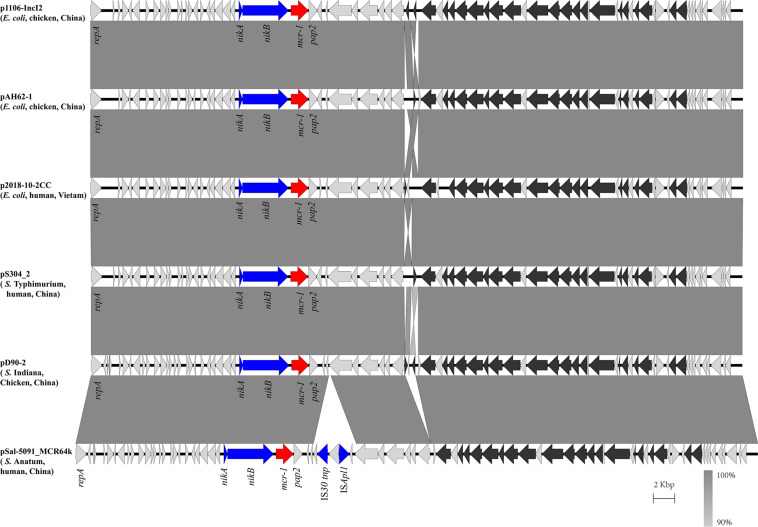
Scaled, linear diagrams comparing the sequences between plasmid pS304_2, p1106-IncI2 (GenBank no. MG825374.1), pAH62-1 (GenBank no. CP055260.1), p2018-10-2CC (GenBank no. LC511622.1), pD90_2 (GenBank no. CP022452.1), and pSal-5091_MCR64k (GenBank no. CP045521.1). Antibiotic resistance genes are indicated in red arrows. The individual conjugation-related genes are indicated with black arrows. Blue arrows denote transposon- and integron-associated genes. Other genes are shown as gray arrows.

**FIGURE 3:**
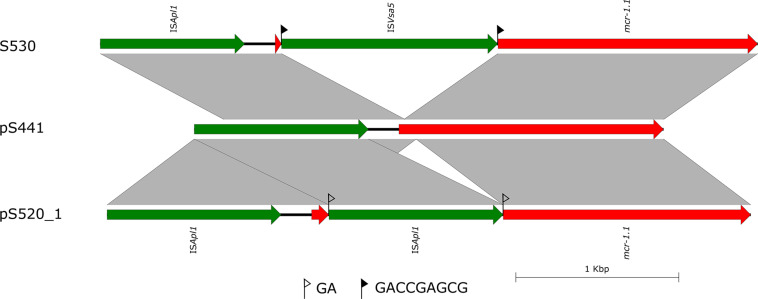
Scaled, linear diagrams comparing the sequences of S530, plasmid pS441, and pS520_1. Insertion elements are shown as green arrows. Genes, including antibiotic resistance genes, are shown as labeled red arrows. Flags indicate the 8 bp target site duplications (TSDs).

In the isolate S304 (a *S.* Typhimurium strain), the *mcr-1* gene was located on IncI2 (60 kb), which was similar to a number of plasmids from different hosts, origins, and regions (Figure 2). The pS304_2 had the simplest *mcr-1* transposon structure (*mcr-1*-*pap2*) without the assistance of the IS*Apl1* gene, as reported previously (Wang J. et al., 2017). These findings confirmed that IncI2-type plasmids have contributed to the successful spread of *mcr-1* gene among species of different diverse genetic environments. Interestingly, in one isolate (*S.* Indiana), the *mcr-1* gene was located in the chromosome. The COL-sensitive isolates S520 and S530 contained *mcr-1* genes that were both disrupted by insertion elements. IS*Apl1* disrupted the *mcr-1* gene in S520, while IS*Vsa5* inserted into the *mcr-1* gene in S530, resulting in non-functional gene products. The identified tandem site duplications (TSDs) with the sequences GA and GACCGAGCG indicate the occurrence of an IS insertion (Figure 3).

### Genetic Environment of the Chromosomal *mcr-1*

**FIGURE 4:**
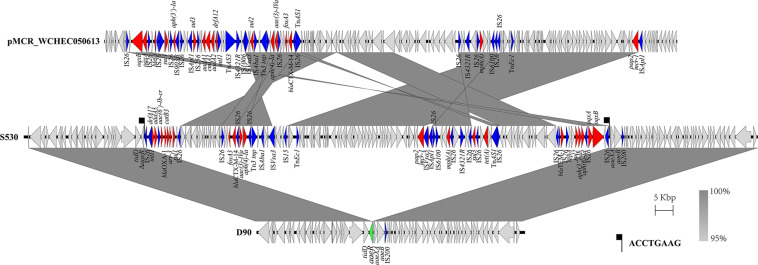
Scaled, linear diagrams comparing the sequences between S530, D90 (GenBank no. CP022450), and pMCR_WCHEC050613 (GenBank no. CP019214). Antibiotic resistance genes are indicated in red arrows. The inserted gene *aaeR* is marked as the green arrow. Blue arrows denote transposon- and integron-associated genes. Other genes are shown as gray arrows.

The genetic arrangement of chromosome-borne *mcr-1* in S530 was investigated. The chromosomal *mcr-1* was adjacent to IS*Apl1* and the *pap2* gene, and they consisted of a “IS*Apl1*-*mcr-1*-*pap2*” structure, which is commonly presented in chromosome-borne *mcr-1* harbored strains (Peng et al., 2019). However, to our surprise, the IncN/IncQ1 replicon and *repA* gene were identified as a sequence surrounding the *mcr-1* gene, indicating a plasmid origin. Bioinformatic analysis showed that a 129.85 kb *mcr-1*-carrying region inserted into the HTH-type transcriptional regulator protein encoding gene *aaeR*, compared with the genome of another *S.* Indiana/ST17 strain D90 (GenBank accession no. CP022450) (Figure 4). This region has a similar genetic environment compared with an IncN/IncHI2 plasmid pMCR_WCHEC050613 (GenBank accession no. CP019214), with 99.99% nucleotide identity at 86% coverage. In addition to the disrupted *mcr-1* gene, this putative plasmid region also harbored multiple resistance genes including *dfrA17*, *aadA5*, *aac(6*′*)-Ib-cr*, *bla*_OXA–1_, *catB3*, *ARR-3*, *sul1*, *fosA3*, *bla*_CTX–M–14_, *aac(3)-IVa*, *aph(4)-Ia*, *sul2*, *floR*, *mph(A)*, *sul3*, *tet(A)*, *bla*_TEM–1B_, *sul2*, *aph(3″)-Ib*, *aph(6)-Id*, *oqxA*, and *oqxB* (Table 3). Further sequence analysis showed that two copies of IS*26* adjacent to the putative plasmid region in same orientation, were flanked by identical 8-bp TSDs (ACCTGAAG), indicating that IS*26* is involved in the insertion of a *mcr-1*-carrying MDR plasmid into the S530 chromosome.

### Transfer Ability of the *mcr-1*-Harboring Plasmids

To evaluate the transferability of the *mcr-1*-harboring plasmids obtained in this study, conjugation experiments were performed. Transconjugants were obtained from each of the mixed cultures of the recipient *E. coli* J53 with one of the five *mcr-1*-positive strains. Four *mcr-1*-positive plasmids were successfully transferred from their hosts via conjugation. The result of antimicrobial susceptibility tests showed that all the positive transconjugants displayed elevated MICs to COL, CTX, and AMP, compared with that of *E. coli* J53 (Supplementary Table 1). It is important to state that since the IncI2 plasmid pS304_2 carried the only antimicrobial resistance *mcr-1*, the co-transferred resistance genes via the IncHI2 plasmid pS304_1 was able to confer the respective antibiotic resistance to the recipients. However, the *mcr-1*-harboring IncHI2 plasmid pS438 failed to transfer to the recipient *E. coli* J53, despite repeating the experiment several times. Further sequence analysis showed that pS438 possessed all the essential genes for mobility, but the transfer-related gene *traG* was disrupted by the insertion element IS*2* (Figure 1), suggesting that the non-transferability of pS438 may be caused by the IS*2* insertion into the *traG* gene.

## Discussion

In 2016, the Chinese Ministry of Agriculture decreed to completely stop the use of COL in animal husbandry for promoting livestock growth and preventing disease (Walsh and Wu, 2016). However, the widespread use of COL in animal farming worldwide over the past decades has resulted in an increase of COL resistance and the spread of *mcr-1*. From 2013 to 2014, the detection rate of pathogenic *E. coli mcr-1* in Japanese pigs was as high as 50% (Kusumoto et al., 2016) and up 21% in French cow dung samples from 2004 to 2014 (Haenni et al., 2016). These findings indicate that the misuse of antibiotics in the livestock industry will greatly increase the selection pressure of bacteria and provide evolutionary advantages for *mcr-1* positive bacteria. As the clinical use of COL has been restricted in the past, the current rate of *mcr-1* positive strains in clinical isolates and in healthy humans is much lower than that in samples that are obtained from farmed animals.

In our study, a total of 689 clinical *Salmonella* strains were screened which were isolated over the past 10 years. Only six of all strains (0.87%) were positive for the *mcr-1* gene. The rate of occurrence of human-derived *Enterobacteriaceae mcr-1* genes has been reported to be less than 2% (Yi et al., 2017a). Lu et al. (2019) collected 12,053 *Salmonella* isolates from a surveillance on diarrhoeal outpatients in Shanghai, China, 2006–2016, and 37 *mcr-1*-harboring strains were detected among them, in which 35 were serovar Typhimurium. In China, the most common ST of *S.* Typhimurium, especially MDR *S.* Typhimurium, is ST34, which is also prevalent in Europe (Antunes et al., 2011; Wong et al., 2013). In this study, all the five plasmid-mediated *mcr-1*-positive *Salmonella* strains belong to *S.* Typhimurium/ST34, suggesting that *mcr-1*-bearing plasmids might have a strong association with specific serotypes of *Salmonella*. Most of the *S.* Typhimurium/ST34 strains carrying *mcr-1* gene were isolated from animals (Yi et al., 2017b), which suggests that the ST34 clone poses a great threat as it is able to disseminate COL resistance from food-producing animals to humans. While the antibiotic is still being used in agriculture (legally or illegally), the last-resort clinical deployment of COL for the treatment of MDR Gram-negative bacteria will lead to an increase in frequency of the *mcr-1* gene, which can be easily transmitted by conjugation. This will aggravate the global antibiotic resistance crisis even further.

Wang Y. et al. (2017) conducted a retrospective analysis of risk factors such as infection, frequency of occurrence and fatality rates of infections by *mcr-1* positive *E. coli* and *K. pneumoniae* in hospitals in Zhejiang and Guangdong, China. The results showed that the status of the immune system as well as the history of antibiotic use (especially carbapenem and fluoroquinolone) are risk factors for infection with *mcr-1* positive strains. In our study, we found that four patients that were infected with *mcr-1* positive strains were young children under 3 years, while the remaining two were 65 and 84 years old, being admitted to the hospital with symptoms of gastroenteritis, gastrointestinal disorders, or diarrhea. This indicated that age and low immune function may have certain effects on *mcr-1* infection. However, due to the small number of patients and *mcr-1* positive strains no statistically significant conclusion can be drawn.

The antimicrobial susceptibility test of the six *mcr-1* positive strains showed that all strains are multidrug resistant, with resistance to many antibiotics, including AK, CRO, FEP, TET, AMC, CTX, and chloramphenicol. Interestingly, only four of the six *mcr-1* positive strains displayed COL resistance. For the two COL-sensitive strains we identified inactive forms of *mcr-1* due to insertion. It has previously been reported that the *mcr-1* gene was inactivated by insertions of either IS*10R* or IS*12984b* (Terveer et al., 2017; Zhou et al., 2018). An inactivation of *mcr-1* by an intragenic 22-bp duplication was described in an isolate of *Shigella sonnei* from Vietnam (Pham Thanh et al., 2016). In our study, we observed two inactive forms of the *mcr-1* gene in *Salmonella*. The *mcr-1* gene was disrupted by IS*Apl1* and IS*Vsa5*, respectively. The strain S520 which harbored the *mcr-1* gene with the insertion of IS*Apl1* was plasmid-encoded in the IncHI2 plasmid pS520_1. As the plasmid also encoded genes mediating resistance to several other antibiotics, the inactive *mcr-1* gene might have been “carried along” on epidemic resistance plasmids. Interestingly, we found one chromosome-encoded *mcr-1* in strain S530, which was, however, disrupted by IS*Vsa5*. So far, a chromosome-embedded *mcr-1* in *Enterobacteriaceae* has been rarely reported (Falgenhauer et al., 2016; Peng et al., 2019). Our bioinformatic analysis showed that the *mcr-1* gene on the chromosome in strain S530 was flanked by IS*Apl1*. Our finding corroborates the hypothesis that the insertion of the *mcr-1* gene into the bacterial chromosome is mediated by IS*Apl1* (Peng et al., 2019). However, in our case, the chromosomal *mcr-1* gene was found to be of plasmid origin. The putative *mcr-1*-carrying IncN/IncQ1plasmid was flanked by two copies of IS*26* that mediated the integration into the *aaeR* gene of the S530. To the best of our knowledge, this study includes the first description of the mobilization of the *mcr-1* gene into a chromosome mediated by a plasmid.

Our study showed that the *mcr-1* gene was encoded by a variety of plasmids, among them IncI2, IncX4 and IncHI2 being the most predominant ones (Sun et al., 2018). Previously, the IncHI2/IncN replicon was reported to carry the *mcr-1* gene, which indicates that the *mcr-1* gene and its surrounding sequence might be derived from the IncHI2/IncN plasmid (Lu et al., 2020). In IncI2 plasmids, *mcr-1-pap2* was the most common, whereas *mcr-1* genes were usually flanked by IS*Apl1* (IS*Apl1-mcr-1-pap2* or Tn*6330*) in IncHI2 plasmids (Cao et al., 2020). This finding is also consistent with the results of our study, with the exception of pS585 that is not embedded in an IS. Although the total positive rate of *mcr-1*-harboring *Salmonella* strains in this study was revealed to be rather low, our conjugation experiments demonstrated that all the *mcr-1*-harboring plasmids are capable of transferring to *E. coli*, except the one plasmid in which *traG* was disrupted by IS*2.* This clearly illustrates the ability of *Salmonella* to spread these plasmids to diverse genera of the *Enterobacteriaceae*. The same, or highly similar, plasmids were also found in various host strains from different sources (animal, food, and humans) isolated in different regions, demonstrating the general applicability of our findings regarding the transfer abilities of the studied *mcr-1*-harboring plasmids among a pool of various microbes, including those that are animal and human pathogens.

Mobile genetic elements, particularly insertion sequence elements (ISs) are able to reorganize the sequence in plasmids. In this study, we observed several IS and transposon insertion events, including those mediated by IS*Apl1*, IS*186B*, IS*103*, IS*2*, and Tn*5403*. Among all the ISs, IS*26* seems to play a major role in the rapid dissemination of antibiotic resistance gene in Gram-negative bacteria (Harmer et al., 2014; He et al., 2015). Partridge et al. (2018) had previously shown that additional IS*26* are easily acquired if the plasmid already possessed a copy of IS*26.* In our study, the insertion of one or more IS*26* mediated several inversion events which involved a plethora of antibiotic resistance genes. IS*26* copies lead to DNA sequence inversion via intramolecular replicative transposition *in trans* (He et al., 2015). This work, as well as our study, demonstrates the importance of replicative transposition in the reorganization of multiple antibiotic resistance plasmids.

In conclusion, we collected 689 clinical *Salmonella* strains and six of them (0.87%) were *mcr-1*-positive. Five strains harbored plasmid-encoded *mcr-1* gene and the other one carried chromosomal *mcr-1* gene originated from plasmid. Five plasmid-mediated *mcr-1*-positive *Salmonella* strains belong to *S.* Typhimurium/ST34 and carried *mcr-1* via two types of plasmids (four IncHI2 plasmids and one IncI2 plasmid). The same or highly similar plasmids were also found in different sources (animal, food, and humans), suggesting that *S*. Typhimurium/ST34 is the potential reservoir for transmission of COL resistance and presents a potential public health threat. Active surveillance of *mcr-1*-harboring *Salmonella*, especially *S*. Typhimurium/ST34, should be further conducted due to the potential high risk of COL resistance development.

## Data Availability Statement

The complete genome sequence of six *mcr-1*-positive *Salmonella* has been deposited in GenBank under accession number CP061115-CP061130.

## Ethics Statement

The studies involving human participants were reviewed and approved by the Ethics Committee of Hangzhou First People’s Hospital (2020103-1). Written informed consent from the participants’ legal guardian/next of kin was not required to participate in this study in accordance with the national legislation and the institutional requirements.

## Author Contributions

JF, YY, and XH designed the study. JF, LZ, JH, and MZ performed the experiments. XH, JH, SL, BL, and LZ analyzed the bioinformatics data. JF, JH, MZ, and XH wrote the manuscript. All authors contributed to the article and approved the submitted version.

## Conflict of Interest

The authors declare that the research was conducted in the absence of any commercial or financial relationships that could be construed as a potential conflict of interest.

## Footnotes

1 https://github.com/tseemann/abricate

2 https://github.com/tseemann/mlst
