# Supplementary material for: Plasmid Dynamics of mcr-1-Positive Salmonella spp. in a General Hospital in China
Source: Front Microbiol. 2020 Dec 22;11:604710. doi: 10.3389/fmicb.2020.604710 (PMC7782425; doi:10.3389/fmicb.2020.604710)
Supplement: Supplementary file 1 [file Image_1.pdf]

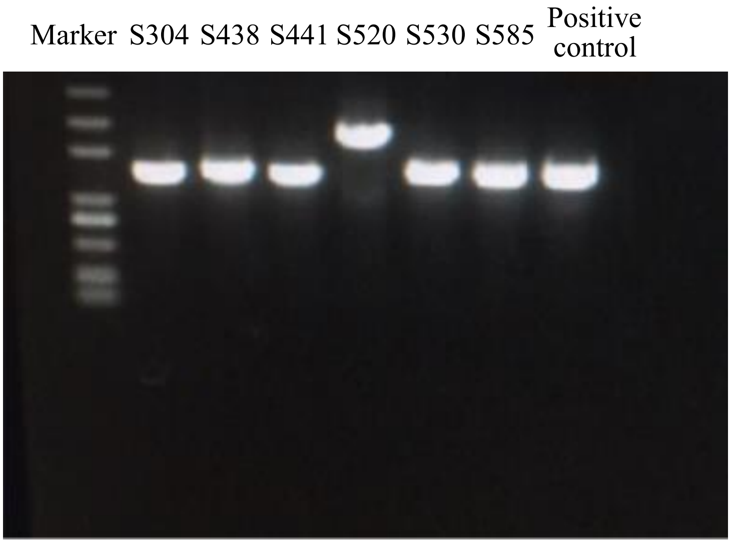

Supplemental Figure 1: PCR electrophoresis map of *mcr-1* positive strains
