# Supplementary material for: Plasmid Dynamics of mcr-1-Positive Salmonella spp. in a General Hospital in China
Source: Front Microbiol. 2020 Dec 22;11:604710. doi: 10.3389/fmicb.2020.604710 (PMC7782425; doi:10.3389/fmicb.2020.604710)
Supplement: Supplementary file 2 [file Image_2.pdf]

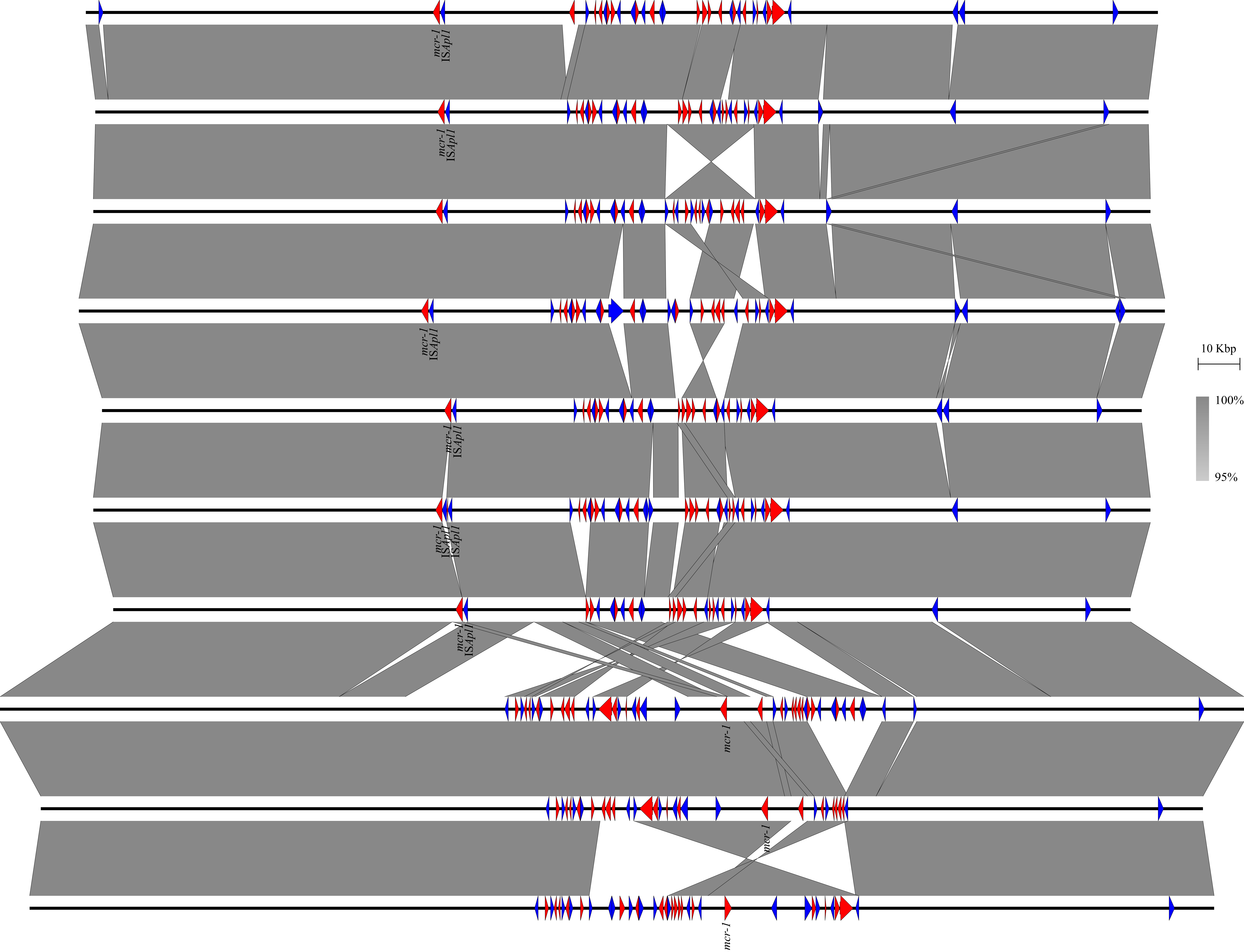

Supplement Figure 2: Scaled, linear diagrams comparing the sequences of plasmid pGDP25-25 (MK673547), pSH15G1428 (MK477605), pS438, pS441, pGDP37-4 (MK673548), pS520, pSH16G0648 (MH522418), pGSJ/2017-Sal-008 (CP050131), pSH16G452 (MH522424) and pS585_1. Antibiotic resistance genes are indicated in red arrows. Blue arrows denote transposon-associated genes.
