# Supplementary material for: Plasmid Dynamics of mcr-1-Positive Salmonella spp. in a General Hospital in China
Source: Front Microbiol. 2020 Dec 22;11:604710. doi: 10.3389/fmicb.2020.604710 (PMC7782425; doi:10.3389/fmicb.2020.604710)
Supplement: Supplementary file 3 [file Table_1.docx]

**Supplemental Table 1: Results of drug sensitivity test of *mcr-1*-positive transconjugants and the recipient *E. coli* J53.**

| Strains | AMK | CTX | COL | TET | AMP |
| --- | --- | --- | --- | --- | --- |
| XH1837 | 8 | 8 | 16 | 8 | >=256 |
| XH1838 | 16 | >=64 | 16 | 2 | >=256 |
| XH1839 | 32 | >=64 | 0.5 | 4 | >=256 |
| XH1840 | 32 | >=64 | 16 | 2 | >=256 |
| J53 | 8 | 0.06 | <=0.03 | 2 | 4 |
| 25922 | 4 | 0.06 | 0.25 | 0.5 | 4 |

Note:

MIC (μg/mL); AMK, amikacin; CTX, cefotaxime; COL, colistin; TET, tetracycline; AMP, ampicillin. The donors of the transconjugant XH1837, XH1838, XH1839, XH1840 were S304, S441, S520, S585, respectively.
